# Home care service employees’ contribution to patient safety in clients with dementia who use dietary supplements: a Norwegian survey

**DOI:** 10.1080/02813432.2021.1970944

**Published:** 2021-09-15

**Authors:** Hilde Risvoll, Frauke Musial, Marit Waaseth, Trude Giverhaug, Kjell Halvorsen

**Affiliations:** a NAFKAM, Department of Community Medicine, UiT, The Arctic University of Norway, Langnes, Tromsø, Norway; b NKS Kløveråsen as, Bodø, Norway; c Valnesfjord Helsesportssenter, Valnesfjord, Norway; d Department of Pharmacy, UiT, The Arctic University of Norway, Langnes, Tromsø, Norway; e RELIS North Norway, University Hospital of North Norway, Tromsø, Norway

**Keywords:** Home care services, dietary supplements, dementia, patient safety, risk management, professional practice behavior, attributed responsibility, cross-sectional survey

## Abstract

**Objective:**

To explore home care services (HCS) employees' professional experiences with the use of dietary supplements (DSs) in their clients with dementia. We also investigated their attributed professional responsibility concerning this use and their attitudes toward DSs in general. Differences between nurses and nurse assistants were investigated.

**Design:**

A cross-sectional survey with self-administered questionnaires.

**Setting:**

Home care services in six Norwegian municipalities in the period August-December 2016.

**Subjects:**

A total of 231 (64% response rate) HCS employees; 78 nurses and 153 nurse assistants (auxiliary nurses and employees without formal education).

**Main outcome measures: **Health care employees’ experiences with patient safety in clients with dementia who use DSs.

**Results:**

Fifty per cent were concerned that clients with dementia might harm their health due to DS use. Thirty-one per cent reported having intervened in order to reduce the risk. Seventy-one per cent preferred to administer DSs to clients with dementia rather than leaving this responsibility to the clients. The respondents placed the responsibility for patient safety in clients with dementia using DSs mainly with the general practitioners, while they ascribed themselves and pharmacies a medium level of responsibility. There were only minor difference between nurses and nurse assistants, and no difference in attitudes towards DSs.

**Conclusion:**

Employees in HCS were concerned about the DS use in clients with dementia. Moreover, almost one-third had intervened to improve clients' patient safety. The majority indicated that HCS should administer DSs rather than the clients with dementia themselves.

**KEY POINTS**
To our knowledge, this is the first study investigating the role of home care services with regard to patient safety in clients with dementia who use dietary supplements (DSs).
•Home care service employees worried about patient safety related to DS use in clients with dementia.
•Home care service employees attributed to themselves medium responsibility to ensure the safe use of DSs in these clients.
•Lack of knowledge was the most important reason why home care service employees did not recommend DSs to clients.

## Introduction

Home care services assist community-dwelling persons (clients) in need of help with their prescription drugs (PDs), nutrition and personal hygiene [1,2]. In Norway, home care services is part of the social welfare system and is provided by local health authorities at the municipality level [3]. Home care service employees have different educational qualifications, some are nurses at the bachelor’s level; however the majority of home care service employees have health-related education at the high school level (auxiliary nurses), and a few are assistants without formal education. All employees, including employees without former education, are allowed to administer PDs from an automated drug-dispensing system and from a prefilled pill organizer to clients after being certified in the control and administration of medication (theoretical and practical education). Some advanced treatments are restricted to those with more education, but for the majority of clients, all types of employees perform the same tasks.

Cognitive impairment, including dementia, is a common reason for receiving assistance from home care services [4,5]. The term dementia covers several diseases that cause a progressive decline in cognitive function and reduce the ability to be self-sufficient in activities of daily living [6].

Up to 57% of persons with dementia use dietary supplements (DSs) [7–9]. The United States Dietary Supplements Health and Education Act (DSHEA) of 1994 defines a DS as a product meant to supplement the diet and includes vitamins, minerals, herbs, botanical products, amino acids, or dietary substances [10]. Generally, people use DSs to improve their health and wellbeing [11]. Although considered natural and safe by many, DSs can compromise health by causing adverse events and/or interact with ongoing PD treatment [12,13] and have also been associated with fatal outcomes [13]. Unapproved pharmaceutical drugs have been found in cognitive enhancement supplements [14]. No specific effect on dementia has been proven so far, even though some single studies may have shown promising results [15–18]. Clients with dementia are at particular risk because their cognitive problems may compromise the correct use of DSs and PDs [7]. Moreover, persons with dementia seldom disclose their DS use to health care personnel [9], leaving general practitioners (GPs) and other health care providers, such as home care services unaware of their use. Dementia symptoms reduce a person’s ability to administer both PDs and DSs correctly, and these clients may therefore need help to administer their PDs [5] and DSs [7].

Home care service employees usually collaborate with GPs and pharmacists to secure safe use of PDs in their clients. In an earlier study, caregivers (next of kin) reported that although home care service employees often assisted clients with dementia with their PDs, they were seldom involved in administering DSs [7].

The aim of this study was to investigate home care service employees’ professional practice, experiences with and knowledge of unsafe DS use in their clients with dementia, including their attitudes towards DSs in general. We also investigated their attribution of responsibility concerning DS use in their clients and investigated differences in professional practice and attitudes between nurses and nurse assistants.

## Methods

### Study population

We conducted a cross-sectional survey between August and December 2016. All home care service employees in six municipalities in Northern Norway were invited to participate. The municipality populations ranged from 1000 to 50,000 inhabitants. We included employees with a minimum of experience working with clients, and excluded employees on long-term sick leave (>8 weeks), employees working less than 40% of the full-time equivalent, employees on temporary employment of less than six months, and administrative personnel. We categorized the respondents into nurses (including social educators and other health-related education at bachelor’s level), nurse assistants (including auxiliary nurses and other health-related education of three years of upper secondary school), and employees without formal education. In the analysis, we combined the latter category with nurse assistants after checking that this did not affect the analysis. The group without formal education was small, and we hypothesized that the largest difference, if any, would be between nurses and assistants in general.

Intermediate leaders assisted in the recruitment of respondents. The response rate was calculated based on the numbers of employees provided by these leaders.

**Figure 1.**
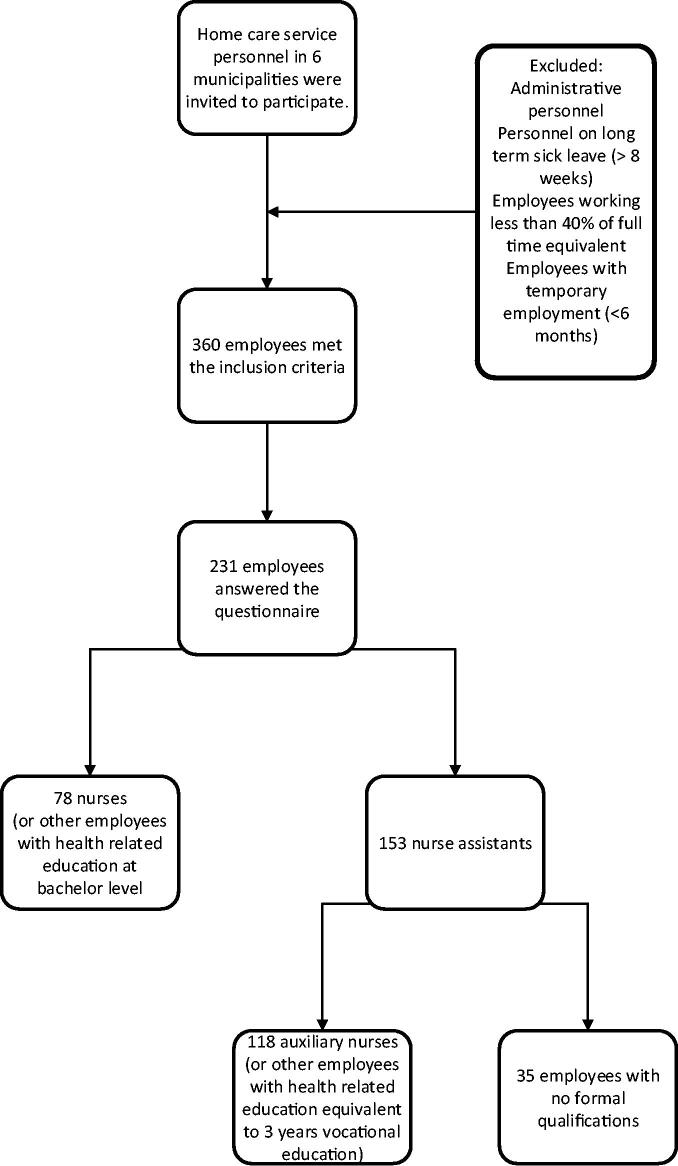
Study population and the recruitment process.

The questionnaire was available both electronically (Questback formula, Questback AS, Oslo, Norway) and in paper format. The home care service employees received three reminders through their intermediate leaders. Figure 1 provides an overview of the study population and the recruitment process.

### Questionnaire

We developed a questionnaire specifically for this study. The questionnaire included 31 questions and took 15–20 min to complete (see Supplementary material 1 (English translation)). A feasibility study testing the questionnaire was conducted by including 15 home care service employees working outside the study area.

The questions about the attribution of responsibilities and suggestions for improvements were ordinal. Respondents were asked to rank the six categories, resulting in a ranking scale from 1–6. We merged scores of 2–4 into a medium-level responsibility/medium preferred category and scores 5–6 into a least-responsibility/least preferred category. In the questionnaire, the term dietary supplements was supplied with natural remedies, but as the definition of DS [10] includes all elements of natural remedies, we only use (the term) DS in the text.

### Ethics

The Regional Committee for Medical and Health Research Ethics had no objections to the study design (2014/1385). The survey did not collect personally identifiable information and was therefore not accountable under the Norwegian Data Protection Agency. All participants were given written information about the study and informed that answering the questionnaire was considered study consent.

### Statistics

We used IBM SPSS (Statistical Package for the Social Sciences) version 23.0 (IBM Corp., Armonk, NY, US) for the statistical analyses. Pearson’s chi-square test or Fisher’s exact test was applied to detect differences in categorical data. *P*-values *<*0.05 were considered statistically significant. Bonferroni correction was performed to correct for potential multiple testing.

## Results

A total of 231 respondents answered the questionnaire, of whom 218 (94%) were women. The response rate was 64% (Figure 1). Seventy-eight (34%) of the respondents were nurses, and 153 (66%) were nurse assistants. Of the respondents, 91 (40%) had 0–5 years of working experience, 74 (32%) had 6–15 years, and 65 (28%) had more than 15 years. One respondent did not answer this question.

Regarding personal use, 143 respondents (62%) used DSs themselves. The majority of the respondents (*n* = 172, 75%) were uncertain whether some DSs could prevent or cure dementia symptoms, 23 (10%) believed some DSs could, and 31 (13%) considered DSs to have no such effects. Five respondents (2%) did not answer this question. The respondents considered the following DSs to be effective against dementia (in descending order): omega-3 fatty acids (*n* = 9) or fish liver oil (*n* = 4), vitamin B12 (*n* = 4), vitamin D (*n* = 3), coconut oil (*n* = 2), calcium (*n* = 1), folic acid (*n* = 1) lactic acid supplement (*n* = 1) and St. John’s wort (*n* = 1). Four respondents indicated that vitamins and minerals could be effective without specifying which vitamins and/or minerals. As a response to the statement ‘Some DSs may pose a threat to users’ health’, 134 respondents (58%) agreed, 15 (6%) disagreed, 73 (32%) were uncertain, and nine (4%) did not answer the question. Eighty (35%) respondents had recommended DSs to clients, of which 71 (31%) had recommended vitamins, 21 (9%) had recommended minerals and three (1%) had recommended herbs, 146 (63%) had not recommended DSs, and 5 (2%) did not answer the question. Among those who recommended DSs (*n* = 80), the following reasons were given: the recommended DS was believed to have scientifically documented effects (*n* = 31, 39%), the DS would not harm the client (*n* = 15, 19%) or the DS would cure or ease symptoms (*n* = 7, 9%). Among those who never had recommended DSs (*n* = 146), the most common reason was lack of knowledge about DSs (*n* = 127, 87%). Other reasons were concern about adverse events and DS-PD interactions (*n* = 58, 40%), recommending DSs was considered beyond their duty (*n* = 50, 34%), DSs were considered ineffective (*n* = 5, 3%) or the clients took enough pills already (*n* = 4, 3%). It was possible to choose more than one reason for recommending or not recommending DSs. There were no differences in reasons for recommending or not recommending DSs between nurses and nurse assistants and no other differences in attitudes between the subgroups (see Supplementary material 2). More employees with longer work experience had recommended DSs than those with less experience. There were no other differences associated with the duration of work experience (see Supplementary material 2).

Table 1 describes how often respondents experienced different professional concerns regarding DSs.

**Table 1.** Professional practice experience related to DS use by clients with dementia.

How often do you, as an employee in home care service,	Several times a week	Weekly-monthly	Monthly-bi-annually	Bi-annually-annually	Annually or less often	Never	Respondents with experience		Differences between nurses and nurse assistants	
	*n*	*n*	*n*	*n*	*n*	*n*	*n*	(%)	*p*	Cramer’s *V*
Fear that clients might suffer harm due to their DS use *n* = 213	3	4	17	17	66	106	107	(50)	0.199	0.184
Experience that caregivers raise concern about clients’ DS use *n* = 222	0	0	1	5	30	186	36	(16)	0.065	0.179
Consult caregivers concerning the safety of clients because of their DS use *n* = 225	0	0	2	9	29	185	40	(18)	0.009	0.229
Experience that clients consult you regarding their DS use *n* = 227	0	0	3	17	51	156	71	(31)	0.347	0.139
Observe DSs in the homes of clients *n* = 226	6	28	20	36	80	56	170	(75)	0.095	0.238
Intervene with clients’ DS use to avoid harm to their health *n* = 224	0	0	1	9	45	169	55	(25)	<0.001*	**0.304**

DS: dietary supplement. The nurse category may include social educators and other health-related education at bachelor’s level. Nurse assistants include auxiliary nurses, other individuals with health-related education (three years of upper secondary school), and employees without formal education. Differences between nurses and nurse assistants were tested with Fisher’s exact test. Bonferroni adjusted *α* was 0.05/6 resulting in *α* = 0.008. Statistically significant differences between subgroups after adjustment are printed in bold and marked with *. A Cramer’s *V* > 0.1 indicate a small effect size, a Cramer’s *V* > 0.3 indicates a medium effect size.

Respondents who had intervened to secure safe DS use by their clients with dementia (*n* = 71) reported which interventions they had performed (Table 2). Of those who had intervened, 59 answered the question about whether they would intervene again, 49 (83%) would (answers a and c, Supplementary material 1), and ten (17%) were more uncertain (answers b and d, Supplementary material 1). As a response to the question about the frequency of interventions, 55 respondents replied that they had intervened at least once (Table 1); however, examining the question about different types of interventions resulted in 71 respondents who reported at least one type of intervention (Table 2). The latter number is reported as the total number of respondents who reported any type of intervention.

**Table 2.** Interventions to increase the safety of clients with dementia who used DSs.

Interventions to increase safety	Respondents		Level of education				Differences between nurses and nurse assistants	
	*n*	(%)	Nurses	Proportions of nurses who applied each intervention	Nurse assistants	Proportions of nurse assistants who applied each intervention	*p*	*Phi*
			*n*	(%)	*n*	(%)		
Consulted GP *n* = 71	32	(45)	22	(28)	10	(7)	<0.001*	**0.431**
Consulted pharmacy *n* = 70	10	(14)	9	(12)	1	(1)	0.002*	**0.363**
Consulted caregiver *n* = 71	19	(27)	10	(13)	9	(6)	0.439	0.092
Asked caregiver to remove DSs *n* = 70	12	(17)	8	(10)	4	(3)	0.109	0.191
Took action to include DSs in automated drug-dispensing system *n* = 70	29	(41)	17	(22)	12	(8)	0.068	0.218
Discussed the problem with colleagues *n* = 70	35	(50)	10	(13)	25	(16)	0.004*	**0.344**

GP: general practitioner; DS: dietary supplement. The nurse category may include social educators and other health-related education at bachelor’s level. Nurse assistants include auxiliary nurses, other individuals with health-related education (three years of upper secondary school), and employees without formal education. Differences between subgroups were tested with Chi-square test. Bonferroni adjusted *α* was 0.05/6 resulting in *α* = 0.008. Statistically significant differences between subgroups after adjustment are printed in bold and marked with *. A phi >0.1 indicates a small effect size, a phi >0.3 indicates a medium effect size.

Concerning who should administer DSs to clients with dementia, 164 respondents (71%) preferred that the home care services performed this service rather than leave the clients to manage by themselves, seven (3%) disagreed, 55 (24%) were uncertain, and five (2%) did not answer the question. To the question "In your opinion, how many of the clients have dementia? Their diagnosis do not need to be confirmed for you to answer", 88 (38%) answered 0–24%, 94 (41%) answered 25–49%, 37 (16%) answered 50–74% and seven (3%) answered 75–100%. Five respondents (2%) did not answer this question.

To the question of whether the employees knew where to find reliable (scientific) information about DSs specified in the questionnaire as ‘not information from the manufacturer or information from magazines or newspapers et cetera’, one-third of the respondents (*n* = 74) confirmed this. The remaining two-thirds either did not know (*n* = 147) or did not respond (*n* = 10). To obtain information or check whether clients’ DSs were safe, the respondents reported consulting GPs (*n* = 14), pharmacies (*n* = 14), the Summary of Product Characteristics (*n* = 4), the internet (*n* = 2), the pharmacovigilance centre (*n* = 2) or the Norwegian Medicines Agency (*n* = 1). Sixty-four respondents (28%) had received information on DSs during their professional training. A minority (*n* = 9, 4%) of the respondents had participated in continuous education on DSs. There was no difference between nurses’ and nurse assistants’ ability to find reliable information on DSs or their view on administering DSs to clients with dementia (see Supplementary material 2).

**Figure 2.**
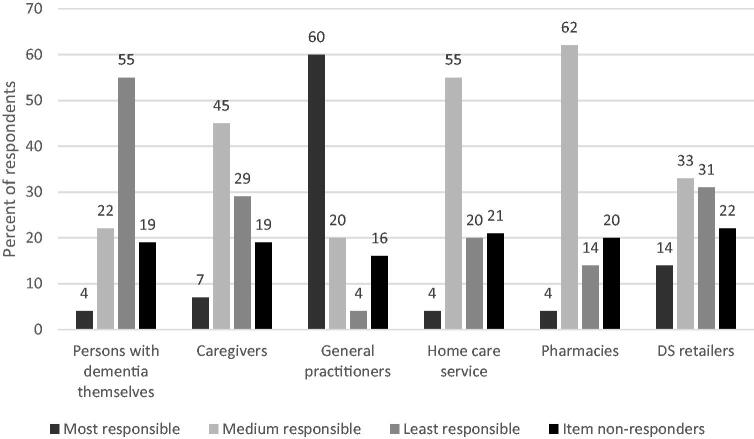
The respondents’ ranking of responsibility for the safety of clients with dementia who use dietary supplements. DS: dietary supplement. DS retailers could be health food store staff, internet retailers, complementary and alternative medicine therapists, or others. For the question ‘Where should the responsibility for the safe use of DS in clients with dementia be placed?’, respondents were asked to rank the six categories from 1 (most responsible) to 6 (least responsible). We merged ranks 2–4 into medium-level responsible and ranks 5–6 into least responsible.

Figure 2 provides an overview of the respondents’ opinion on who should be responsible for the safe use of DSs in clients with dementia. GPs were considered most responsible, and there were no differences between subgroups (see Supplementary material 2).

**Figure 3.**
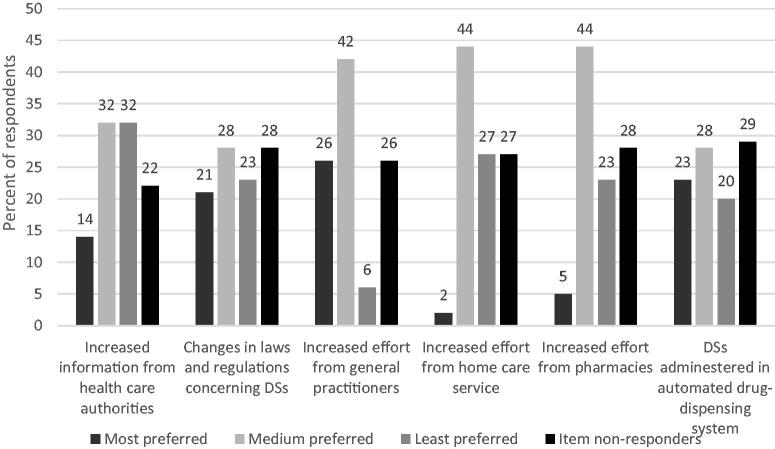
Respondents’ opinions on how to improve the safety of clients with dementia who use dietary supplements. DS: dietary supplement. The employees were given six alternatives on how to ensure the correct and safe use of DSs. Respondents were asked to rank the six categories from 1 (most preferred) to 6 (least preferred). We merged priorities 2–4 into medium-level priority and priorities 5–6 into lowest-priority.

Figure 3 provides an overview of the respondents’ opinions on how to improve the safety of clients with dementia who use DSs. Most respondents chose increased effort from GPs as the most preferred intervention, followed by DSs administered *via* the automated drug-dispensing system and changes in laws and regulations concerning DSs. The only difference between health care personnel groups was that nurses were less positive about the suggestion to administer DSs *via* the automated drug-dispensing system as the most preferred option (see Supplementary material 2).

## Discussion

### Statement of principal findings

Half of the respondents were worried about potentially harmful DS use in clients with dementia, and almost one-third had intervened to secure safety. Most of those who had intervened would do it again. Nurses’ and nurse assistants’ interventions differed according to their professional responsibilities; however, their attitudes towards DSs were similar. The respondents did not consider themselves as primarily responsible for patient safety in clients with dementia who use DSs but attributed this responsibility to the GPs. A minority had received education on DSs.

### Strengths and weaknesses

The major strength of this study is its originality. There are very few studies among home care services in general, and we have not identified any other study exploring this particular topic. We invited all home care service employees who had sufficiently recent professional experience with clients to maintain external validity. The response rate was satisfactory, and the total number of respondents was comparable to related studies [19,20].

The study included a high proportion of nurse assistants. Nurse assistants make up a substantial proportion of employees in Norwegian home care services, and their experiences and attitudes are highly relevant for clients. The person with dementia receiving services from home care services does not necessarily know whether it is a certified nurse who is visiting or a person without formal education, as the professional tasks in most cases are the same. All groups of employees, including the group without formal education, had experience with different aspects of worrying or counselling regarding clients’ use of DSs.

The team behind this study has a multidisciplinary background, including experience from a dementia clinic, pharmacological expertise, user-expertise and expertise on complementary and alternative medicine. We believe this increased the quality and relevance of the survey questions and the interpretation of the results.

The results should be generalizable for Norway and areas with similar health care systems, such as Scandinavian countries. Nevertheless, we believe the study findings are relevant for home care services or nurses caring for people with dementia in their homes regardless of country of residence.

Weaknesses of the study include that some of the questions had a high proportion of item nonrespondents. This mainly applies to the question about attribution of responsibility, which might be difficult to answer, as it also relates to the organization of the health care system. Another question with high nonresponse concerned reasons for recommending DSs, which might have been better captured by an open-ended question. Furthermore, we cannot totally exclude either selection bias or recall bias.

Lack of time could be an important reason for not noticing problems related to DSs. We did not include a question about how many visits/clients each respondent attended to per shift, but in retrospect asked their intermediate leaders about this. They estimated the number of visits per shift to vary between eight and 20, which could include several visits to the same clients. The visits took from ten minutes to several hours. We have no reliable knowledge on how many clients with dementia these employees visited every day/week.

### Findings in relation to other studies

Unsafe and inappropriate use of PDs has been reported in another Norwegian home care service setting [21], where unclear documentation and adverse events were more prominent among home care service clients (*n* = 93) than among nursing home residents (*n* = 61). We have not identified any other study exploring home care service employees’ contribution to securing patient safety in clients with dementia who use DS, their awareness of the problem and attribution of responsibility.

We previously conducted a similar study among employees in pharmacies in the same geographical area [22]. In contrast to the high proportion of home care service employees who reported worries about unsafe use of DSs in their clients, only 8% of employees in pharmacies reported similar worries. Home care service employees are closer to their clients than pharmacy employees; they visit their clients multiple times in their homes and are to a greater extent aware of their clients’ cognitive capacity than pharmacy employees. Attitudes towards the safety and efficiency of DSs were similar between home care service employees and pharmacy employees, as approximately ten percent of the respondents in both study populations believed in effects derived from DSs in the treatment of dementia and approximately 60% agreed with the statement that DSs in some cases can compromise users’ health. Likewise, 35% of both study populations had recommended DSs to clients [22].

In this study, we did not investigate actual DS use in clients. In a previous study, we revealed that 46% of patients with dementia (*n* = 151) used DSs, and on average, these patients used 1.7 DSs [7]. Fish oils were the most commonly used DS (57%), followed by various mixed herbal supplements (41%) and vitamin and mineral supplements (40%). We identified potentially clinically relevant interactions between DSs and PDs in 11% of DS users, which was mainly due to the use of herbs.

It needs to be emphasized that DSs constitute a very large and diverse group of products in which some, such as herbs, are more prone to cause adverse events and interactions. Vitamins, minerals and fish oils are also defined as DSs, and although these may be a part of medical doctors’ prescriptions [23], most are bought over-the-counter. They are less prone to interactions than herbals, but fat-soluble vitamins may accumulate and cause toxic reactions.

Lack of knowledge is a barrier to communication about complementary and alternative medicine [24,25]. Health care personnel who possess such knowledge are more likely to discuss issues related to DSs with clients [25] and may therefore be better equipped to reveal unsafe DS use in clients. Health care professionals have stated that being trained on DSs is essential, and lack of training raises ethical implications in performing their professional tasks [26]. We did not address knowledge of DSs in our study and have not identified other studies exploring home care service employees’ knowledge of DSs. However, only one out of four respondents had received training on DSs during their education and almost none had received continuing education on DSs. Lack of knowledge was the main reason why the respondents did not recommend DS to their clients, as seen in another study regarding complementary and alternative methods in general [25]. Even though one-third of the respondents claimed to know where to find reliable information about DSs, we did not know if this was the case because the study design only explored the respondents’ opinions on this matter. The factual proportion could be smaller.

The respondents did not consider themselves to have the main responsibility for patient safety in clients with dementia who use DS. Instead, they placed this responsibility with the GPs. This corresponds with the results of a homologous survey among pharmacy employees [22]. Potential reasons could be lack of knowledge of DS contents and safety profiles and concerns about the effects of DSs in frail, older, polymedicated people. Moreover, it is not always known to home care service employees or pharmacy employees whether their clients have dementia. Even if the majority of the respondents in this study believed that less than half of their clients had dementia, underdiagnosis of dementia is common also in the home care service setting [4,27]. Concerning suggestions for improvements, the main difference between this study and a homologous study among pharmacy employees [22] is that home care service employees were more positively oriented to include DSs in the automated drug-dispensing system. Nurses were less positive than nurse assistants, which might relate to their understanding of drug treatment and limitations in the automated drug-dispensing system (see Supplementary material 2). In both studies, a multidisciplinary approach (i.e. between home care services, pharmacies and especially GPs) was considered necessary for securing patient safety in clients who use DSs.

### Implications

The study implies that home care services have the potential to play a role in securing patient safety in clients with dementia who use DSs. No guidelines or regulations are in place regarding healthcare professionals’ responsibility for safe DS use in their clients [28]. This lack of clear responsibilities compromises patient safety in clients with cognitive impairment. We suggest that a collaboration between GPs, home care services and pharmacy employees that also includes caregivers is the best way to secure safe DS use in clients who are incapable of handling this themselves due to dementia, similar to routines for safe use of PDs [29]. The first step to safeguard DS use is to identify it. Home care service employees have a unique position, as they perform regular home visits. To some degree, they already uncover such problems today, although it is not a systematic part of their job routine. Home care services could communicate findings to GPs and ask pharmacists for advice. If the use is safe and to be continued, an evaluation is needed to assess whether the client with dementia is capable of self-administering. If not, home care services might help with the administration as the majority of the respondents agreed to. Pharmacies and GPs will be involved in including DSs in the automated drug-dispensing system.

Longitudinal observational studies are needed to establish the true frequency of unsafe DS use in clients with dementia in the home care service setting. Such studies should include DS-PD interaction analyses. Moreover, to identify barriers that home care service employees experience when assisting clients with dementia who use DSs, a qualitative study methodology is favourable.

In the Norwegian home care service, the number of nurse assistants is greater than the number of nurses. There were some differences between nurses and nurse assistants in professional conduct related to DS safety. Nurses intervened more often than nurse assistants and communicated more often with other health care professionals, such as GPs and pharmacists to increase the safety of their clients. Nurse assistants discussed problems at work to a greater extent than nurses. This could be explained by different professional roles and responsibilities, where nurse assistants might find it natural to seek advice from nurses/other colleagues in difficult work-related situations. Most importantly, there were no differences in awareness of the problem and feelings of responsibility or in attitudes towards DSs.

The respondents did not report their worries about DS use in clients with dementia to occur frequently. This could indicate that unsafe DS use is an infrequent problem or that there has been little or no focus on discovering such problems. Our data reveal that only a minority of the employees had received education on DSs. We believe that more focus on the safety of DS use in persons with dementia, including an increased focus on education of home care service employees on DSs, is needed. It is important for employees to possess evidence-based knowledge about common DSs to give advice to clients, and especially to know which DSs need to be checked for interactions.

## Supplementary Material

Supplemental Material

Supplemental Material
