# Supplementary material for: Home care service employees’ contribution to patient safety in clients with dementia who use dietary supplements: a Norwegian survey
Source: Scand J Prim Health Care. 2021 Sep 15;39(4):403–12. doi: 10.1080/02813432.2021.1970944 (PMC8725956; doi:10.1080/02813432.2021.1970944)
Supplement: Supplemental Material [file IPRI_A_1970944_SM9511.docx]

## Supplementary material 1. Questionnaire: home care service (translated)

1. Gender?
   1. Female
   2. Male
2. Education?
   1. Nurse (including social educators or others with health-related educational programs at bachelor’s level)
   2. Auxiliary nurse (includes other types of health-related education from vocational school (three years))
   3. No education or no health-related education
3. For how long have you worked in home care service (HCS)?
   1. 0-5 years
   2. 6-15 years
   3. More than 15 years
4. Do you use some of the following dietary supplements (DSs)/natural remedies? You can give more than one answer.
   1. Vitamins
   2. Minerals
   3. Herbs
   4. Other types of DSs/natural remedies
   5. I do not use DSs/natural remedies
5. Do you believe some DSs/natural remedies can prevent or cure dementia symptoms?
   1. Yes
   2. No
   3. I do not know
6. Which DSs/natural remedies do you believe can prevent or cure dementia symptoms?
7. Have you recommended DSs/natural remedies to clients? You can give more than one answer.
   1. I have never recommended DSs/natural remedies
   2. I have recommended vitamins
   3. I have recommended minerals
   4. I have recommended herbs
   5. I have recommended other types of DSs, includes homeopathic medicine
8. If you never have recommended DSs/natural remedies to clients, why not? You can give more than one answer.
   1. I do not have sufficient knowledge about DSs/natural remedies to recommend
   2. I do not believe DSs/natural remedies to have positive effects
   3. The risk of adverse events and interactions with prescribed modifications (PDs)
   4. My clients take enough tablets as it is
   5. It is not my job to recommend
9. If you have recommended DSs/natural remedies to clients, what were your reasons for recommending? You can give more than one answer.
   1. The types of DSs/natural remedies I recommend have positive effects (scientifically documented effects)
   2. I believe the DSs/natural remedies will cure or ease the clients’ symptoms
   3. I believe DSs/natural remedies to be harmless at least
10. In your opinion, how many of the clients have dementia? Their diagnosis do not need to be confirmed for you to answer.
    1. 0-24%
    2. 25-49%
    3. 50-74%
    4. 75-100%
11. How many of your clients have a confirmed dementia diagnosis documented in the clients’ electronic health records?
    1. 0-24%
    2. 25-49%
    3. 50-74%
    4. 75-100%
12. How many of your clients use DSs/natural remedies without receiving help with the administration?
    1. 0-24%
    2. 25-49%
    3. 50-74%
    4. 75-100%
13. How often do you meet clients with dementia that you fear might harm their health, due to their use of DSs/natural remedies? By this, we mean that the DSs/natural remedies can have unfortunate health effects either because of direct toxic effects or because the clients are incapable of correct administration.
    1. Never
    2. Annually or less often
    3. Bi-annually to annually
    4. Monthly to bi-annually
    5. Weekly to monthly
    6. Several times a week
14. How often do your clients’ caregivers (next of kin) discuss their worries about the clients’ use of DSs/natural remedies with you?
    1. Never
    2. Annually or less often
    3. Bi-annually to annually
    4. Monthly to bi-annually
    5. Weekly to monthly
    6. Several times a week
15. Regarding worry about DSs/natural remedy use among clients with dementia, how often do you discuss such worries with client’s caregiver/next of kin?
    1. Never
    2. Annually or less often
    3. Bi-annually to annually
    4. Monthly to bi-annually
    5. Weekly to monthly
    6. Several times a week
16. How often do clients with dementia ask you for advice about their DSs/natural remedies?
    1. Never
    2. Annually or less often
    3. Bi-annually to annually
    4. Monthly to bi-annually
    5. Weekly to monthly
    6. Several times a week
17. How often have you observed DSs/natural remedies lying about in the homes of your clients with dementia?
    1. Never
    2. Annually or less often
    3. Bi-annually to annually
    4. Monthly to bi-annually
    5. Weekly to monthly
    6. Several times a week
18. How often have you interfered when your clients with dementia used DSs/natural remedies with the intention to increase the clients’ safety? Either because you feared, the DS/natural remedy products themselves could cause harm to the clients’ health, or because you feared, the clients did not manage to administer the DS/natural remedy products correctly by themselves.
    1. Never
    2. Annually or less often
    3. Bi-annually to annually
    4. Monthly to bi-annually
    5. Weekly to monthly
    6. Several times a week
19. This question does only apply for those who answered question 18 b-f. How did you interfere? You can give more than one answer.
    1. Consulted the clients’ caregivers (next of kind)
    2. Consulted the clients’ general practitioners (GPs)
    3. Consulted a pharmacy (pharmacists)
    4. Asked the clients’ caregivers (next of kin) to remove the DS/DSs
    5. Arranged for the DSs to be administered by HCS after a GP or a pharmacists had checked that the products were safe to use (did not interact with the clients’ prescribed medications (PDs))
    6. Discussed the safety issue with my colleagues at work
20. This question does only apply for those who answered question 18 b-f. If you have interfered one or several times and later experienced that your interference did not lead to any improvements for your clients, did this make you stop interfering?
    1. I have interfered and experienced an improvement in clients’ safety
    2. I have stopped interfering because my interference did not lead to any improvements for my clients
    3. I have not stopped interfering even though earlier attempts did not lead to any improvements for my clients
    4. I am uncertain if I will interfere again
21. Do you prefer the clients with dementia who use DSs/natural remedies to have their DSs/natural remedies administered by HCS rather than managing the administration by themselves?
    1. Yes
    2. No
    3. I do not know
22. If HCS should be responsible for supervising DSs/natural remedies use by new clients with dementia, would that be problematic? You can give more than one answer.
    1. No, I already do this
    2. I do not find this responsibility problematic, even though I am not responsible for this today
    3. Yes, because of time. I would not have enough time to take on more responsibilities
    4. Yes, because of ethical considerations, it is not ethically right to ask the clients about this
    5. Practical problems would make this responsibility problematic
23. This question is only applicable for those who answered 22e. What practical problems would make this responsibility problematic?
24. Have you received information about DSs/natural remedies during your professional training?
    1. Yes
    2. No
    3. I have no professional training
25. Have you participated in continuous education on DSs/natural remedies after you started working in HCS?
    1. Yes
    2. No
26. Do you know where to find scientific information about DSs/natural remedies products? (not information from the manufacturer or information from magazines or newspapers et cetera)
    1. Yes
    2. No
27. Where do you get the best information about DSs/natural remedies?
    1. Pharmacies/pharmacists
    2. GPs
    3. Other employees in HCS
    4. Pharmacovigilance center
    5. I do not know
28. If you as a part of your professional work have checked whether clients’ DSs/natural remedies were safe, how did you do this? If you have never done this, please write, “Not applicable”.
29. Do you agree with this statement:” DSs/natural remedies may pose a threat to users’ health”?
    1. Yes
    2. No
    3. I do not know
30. Which options are most adequate to secure safe use of DSs/natural remedies by persons with dementia? Please prioritize the options from one to six (one as most adequate, two as second most adequate and so forth).
    1. Information from the health authorities to the general population
    2. Changes in laws and regulations concerning DSs/natural remedies (indicates increased control with the DS content such as increased testing for toxic effects. As today, DSs has less strict safety routines compared to PDs)
    3. Increased effort from GPs (ask all patients about DS/natural remedy use and check for adverse events and interactions)
    4. Increased effort from HCS (ask all clients about DS/natural remedy use and convey information about use to GPs or pharmacists)
    5. Increased effort from pharmacies/pharmacists (for all customers who buy DSs/natural remedies, check for interactions, and inform GPs and, if appropriate, HCS about findings when interactions are identified)
    6. DSs/natural remedies delivered in automated drug-dispensing systems together with PDs (when it has been established that DS is safe to use)
31. Who should be responsible for the correct and safe use of DSs/natural remedies by persons with dementia? Please prioritize the options from one to six (one as most responsible, two as second most responsible, and so forth).
    1. The person with dementia him/her-self
    2. The caregivers
    3. The retailers of DSs/natural remedies
    4. The pharmacies/pharmacists
    5. The GPs
    6. HCS
