# Supplementary material for: Home care service employees’ contribution to patient safety in clients with dementia who use dietary supplements: a Norwegian survey
Source: Scand J Prim Health Care. 2021 Sep 15;39(4):403–12. doi: 10.1080/02813432.2021.1970944 (PMC8725956; doi:10.1080/02813432.2021.1970944)
Supplement: Supplemental Material [file IPRI_A_1970944_SM9510.docx]

# Supplementary material 2

## Suppl. Table 1a. Attitudes towards DS. Nurses versus nurse assistants.

|  | | Nurses | | | Nurse assistants | | χ^2^ /degrees of freedom | *P* |
| --- | --- | --- | --- | --- | --- | --- | --- | --- |
|  |  | n | (%) | | n | (%) |  |  |
| Personal use of DSs  n=143 | | 52 | | (67) | 92 | (60) | 1.199 (1) | 0.274 |
| Believed certain DSs could prevent or cure dementia symptoms  n=23 | | 11 | | (14) | 12 | (8) | 2.806 | 0.415† |
| Agreed that DSs might pose a threat to users’ health  n=134 | | 53 | | (69) | 81 | (53) | 6,021 (3) | 0.110 |
| Preferred their clients with dementia to have their DSs administered by the HCS rather than leave them to manage the administration by themselves  n=164 | | 56 | | (72) | 108 | (70) | 6.486 | 0,067† |
| Have recommended DSs to clients  n=80 | | 31 | | (40) | 49 | (32) | 0.983 (1) | 0.321 |
| Reasons for recommending DSs | | | | | | | | |
|  | Positive documented effects  n=31 | 14 | | (18) | 17 | (11) | 3.176 (1) | 0.075 |
|  | The DSs caused no harm to clients  n=15 | 3 | | (4) | 12 | (8) | 2.100 (1) | 0.147 |
|  | The DSs would cure or ease symptoms  n=7 | 23 | | (3) | 5 | (3) |  | 1.000† |
| Bonferroni adjusted α was 0.05/3 resulting in α=0.017. | | | | | | | | |
| Reasons for not recommending DSs | | | | | | | | |
|  | Lack of knowledge about DSs  n=127 | 42 | | (54) | 85 | (56) | 0.051 (1) | 0.822 |
|  | Concern about adverse events and DS-PD interactions  n=58 | 26 | | (33) | 32 | (22) | 5.086 (1) | 0.024 |
|  | Recommending DSs was considered beyond their duty  n=50 | 12 | | (15) | 38 | (25) | 2.910 (1) | 0.088 |
|  | DSs were considered ineffective  n=5 | 1 | | (1) | 4 | (3) |  | 0.665† |
|  | The clients took enough tablets already  n=4 | 1 | | (1) | 3 | (2) |  | 1.000† |
| Bonferroni adjusted α was 0.05/5 resulting in α=0.01. | | | | | | | | |

DS, dietary supplement. HCS, home care service. PD, prescription drug. The nurse category may include social educators and other health-related education at bachelor’s level. Nurse assistants include auxiliary nurses, other individuals with health-related education (three years of upper secondary school), and employees without formal education. Differences between subgroups were tested with Chi-square test or Fisher exact test †.There were no statistically significant differences between subgroups.

## Suppl. Table 1b. Attitudes towards DS. Years of work experience.

|  | | 0-5 years’ experience | | | 6-10 years’ experience | | >15 years’ experience | | χ^2^ /degrees of freedom | *P* |
| --- | --- | --- | --- | --- | --- | --- | --- | --- | --- | --- |
|  |  | n | (%) | | n | (%) | n | (%) |  |  |
| Personal use of DSs  n=143 | | 54 | | (61) | 45 | (63) | 44 | (69) |  | 0.721† |
| Believed certain DSs could prevent or cure dementia symptoms  n=23 | | 11 | | (12) | 10 | (14) | 2 | (3) |  | 0.415† |
| Agreed that DSs might pose a threat to users’ health  n=134 | | 52 | | (58) | 45 | (63) | 36 | (56) |  | 0.972† |
| Preferred their clients with dementia to have their DSs administered by the HCS rather than leave them to manage the administration by themselves  n=164 | | 64 | | (72) | 51 | (71) | 49 | (77) |  | 0,627† |
| Have recommended DSs to clients  n=80 | | 23 | | (26) | 21 | (29) | 35 | (54) |  | <0.001† |
| Reasons for recommending DS | | | | | | | | | | |
|  | Positive documented effects  n=31 | 10 | | (11) | 5 | (7) | 16 | (25) | 2.482 (2) | 0.289 |
|  | The DS caused no harm to clients  n=15 | 8 | | (9) | 3 | (4) | 4 | (6) | 2.727 (2) | 0.256 |
|  | The DS would cure or ease symptoms  n=7 | 2 | | (3) | 2 | (3) | 3 | (5) |  | 0.875† |
| Bonferroni adjusted α was 0.05/3 resulting in α=0.017. | | | | | | | | | | |
| Reasons for not recommending DS | | | | | | | | | | |
|  | Lack of knowledge about DSs  n=127 | 56 | | (63) | 41 | (57) | 30 | (47) | 1.531 (2) | 0.465 |
|  | Concern about adverse events and DS-PD interactions  n=58 | 20 | | (23) | 22 | (31) | 16 | (25) | 2.115 (2) | 0.347 |
|  | Recommending DSs was considered beyond their duty  n=50 | 15 | | (17) | 20 | (28) | 15 | (23) | 4.338 (2) | 0.114 |
|  | DSs were considered ineffective  n=5 | 4 | | (5) | 1 | (1) | 0 | 0 |  | 0.295† |
|  | The clients took enough tablets already  n=4 | 0 | | (0) | 4 | (6) | 0 | (0) |  | 0.016† |
| Bonferroni adjusted α was 0.05/5 resulting in α=0.01. | | | | | | | | | | |

DS, dietary supplement. HCS, home care service. PD, prescription drug. Differences between subgroups were tested with Chi-square test or Fisher exact test †. There were no statistically significant differences between subgroups.

## Suppl. Table 2a. Respondents access to knowledge on DSs. Nurses versus nurse assistants

|  | Nurses | | Nurse  assistants | | χ^2^ /degrees of freedom | *P* |
| --- | --- | --- | --- | --- | --- | --- |
|  | n | (%) | n | (%) |  |  |
| Knew where to find reliable (scientific) information aboutDSs  n=74 | 27 | (35) | 47 | (31) | 2,774 (2) | 0,250 |
| Received information on DSs during their professional training  n=64 | 23 | (30) | 41 | (27) | 0,090 (1) | 0,764^α^ |
| Participated in continuous education on DSs after they started working in HCS  n=9 | 3 | (4) | 6 | (4) | 0.665 | 0,830† |

DS, dietary supplement. HCS, home care service. The nurse category may include social educators and other health-related education at bachelor’s level. Nurse assistants include auxiliary nurses, other individuals with health-related education (three years of upper secondary school), and employees without formal education. Differences between subgroups were tested with Chi-square test or Fisher exact test †. ^α^ We excluded 22 respondents who had not studied when analyzing this question. There were no statistically significant differences between subgroups.

## Suppl. Table 2b. Respondents access to knowledge on DS. Years of work experience

|  | 0-5 years’ experience | | 6-15 years’ experience | | >15 years’ experience | | χ^2^ /degrees of freedom | *p* |
| --- | --- | --- | --- | --- | --- | --- | --- | --- |
|  | n | (%) | n | (%) | n | (%) |  |  |
| Knew where to find reliable (scientific) information about DSs  n=74 | 34 | (38) | 24 | (33) | 16 | (25) |  | 0,449† |
| Received information on DSs during their professional training  n=64 | 32 | (36) | 19 | (26) | 12 | (19) |  | 0,018^α^† |
| Participated in continuous education on DSs after they started working in HCS  n=9 | 5 | (6) | 2 | (3) | 2 | (3) |  | 0,833† |

DS, dietary supplement. HCS, home care service. Differences between subgroups tested with Chi-square test or Fisher exact test †. ^α^ We excluded 22 respondents who had not studied when analyzing this question. There were no statistically significant differences between subgroups

## Suppl. Table 3a. Employees’ opinions on how to improve the safety of clients with dementia who use DSs. Nurses versus nurse assistants.

|  |  | Nurses | Nurse assistants | χ^2^ /degrees of freedom | *P* |
| --- | --- | --- | --- | --- | --- |
| Increased information from health care authorities | | | | | |
|  | Most preferred | 7 | 27 |  |  |
|  | Medium preferred | 24 | 50 | 6.958 (2) | 0.031 |
|  | Least preferred | 33 | 39 |  |  |
|  | Item non-responders | 14 | 37 |  |  |
| Changes in laws and regulation | | | | | |
|  | First priority | 19 | 30 |  |  |
|  | Medium priority | 20 | 45 | 1.238 (2) | 0.538 |
|  | Last priority | 21 | 32 |  |  |
|  | Item non-respondents | 18 | 46 |  |  |
| Increased effort from GPs | | | | | |
|  | First priority | 27 | 34 |  |  |
|  | Medium priority | 32 | 66 | 2.392 (2) | 0.303 |
|  | Last priority | 4 | 9 |  |  |
|  | Item non-respondents | 15 | 44 |  |  |
| Increased effort from HCS | | | | | |
|  | First priority | 2 | 3 |  |  |
|  | Medium priority | 39 | 61 | 1.483 (2) | 0.530 |
|  | Last priority | 19 | 44 |  |  |
|  | Item non-respondents | 18 | 45 |  |  |
| Increased effort from pharmacies | | | | | |
|  | First priority | 3 | 10 |  |  |
|  | Medium priority | 38 | 63 | 1.059 (2) | 0.589 |
|  | Last priority | 19 | 34 |  |  |
|  | Item non-respondents | 18 | 46 |  |  |
| DSs administered in automated drug-dispensing system | | | | | |
|  | First priority | 13 | 40 |  |  |
|  | Medium priority | 34 | 31 | 12.496 (2) | **0.002*** |
|  | Last priority | 12 | 34 |  |  |
|  | Item non-respondents | 19 | 48 |  |  |

DS, dietary supplement. GP, general practitioner. HCS, home care service. The nurse category may include social educators and other health-related education at bachelor’s level. Nurse assistants include auxiliary nurses, other individuals with health-related education (three years of upper secondary school), and employees without formal education. Differences between subgroups were tested with Chi-square test. Bonferroni adjusted α was 0.05/6 resulting in α=0.008. Statistically significant differences between subgroups after adjustment are printed in bold and marked with *.

## Suppl. Table 3b. Employees’ opinions on how to improve the safety of clients with dementia who use DSs. Years of work experience.

|  |  | 0-5 years’ experience | 6-15 years’ experience | >15 years’ experience | *P* |
| --- | --- | --- | --- | --- | --- |
| Increased information from health care authorities | | | | | |
|  | Most preferred | 13 | 9 | 12 |  |
|  | Medium preferred | 28 | 29 | 16 | 0,563 |
|  | Least preferred | 31 | 18 | 23 |  |
|  | Item non-responders | 19 | 18 | 14 |  |
| Changes in laws and regulation | | | | | |
|  | First priority | 19 | 19 | 10 |  |
|  | Medium priority | 28 | 20 | 17 | 0.259 |
|  | Last priority | 24 | 11 | 18 |  |
|  | Item non-respondents | 20 | 24 | 20 |  |
| Increased effort from GPs | | | | | |
|  | First priority | 19 | 18 | 24 | 0.390 |
|  | Medium priority | 44 | 31 | 22 |  |
|  | Last priority | 5 | 6 | 2 |  |
|  | Item non-respondents | 23 | 19 | 17 |  |
| Increased effort from HCS | | | | | |
|  | First priority | 2 | 1 | 2 | 0.357 |
|  | Medium priority | 39 | 28 | 32 |  |
|  | Last priority | 25 | 27 | 11 |  |
|  | Item non-respondents | 25 | 18 | 20 |  |
| Increased effort from pharmacies | | | | | |
|  | First priority | 6 | 4 | 3 | 0.641 |
|  | Medium priority | 40 | 32 | 29 |  |
|  | Last priority | 20 | 21 | 11 |  |
|  | Item non-respondents | 25 | 17 | 22 |  |
| DSs administered in automated drug-dispensing system | | | | | |
|  | First priority | 19 | 13 | 21 | 0.131 |
|  | Medium priority | 27 | 25 | 13 |  |
|  | Last priority | 16 | 19 | 10 |  |
|  | Item non-respondents | 29 | 17 | 21 |  |

DS, dietary supplement. GP, general practitioners. HCS, home care service. Years of work experience were given in the ranges: 1-5 years’ experience, 6-15 years’ experience and >15 years’ experience. Differences between subgroups were tested with Fisher exact test. Bonferroni adjusted α was 0.05/6 resulting in α=0.008. There were no statistically significant differences between subgroups.

## Suppl. Table 4a. The respondents' ranking of responsibility for the safety of clients with dementia who use DSs. Nurses versus nurse assistants.

|  |  | Nurses | Nurse assistants | χ^2^  (degrees of freedom) | *P* |
| --- | --- | --- | --- | --- | --- |
| Persons with dementia themselves | | | | | |
|  | Most responsible | 1 | 8 |  |  |
|  | Medium | 19 | 36 | 2.778 (2) | 0.249 |
|  | Least | 49 | 77 |  |  |
|  | Item non-responders | 9 | 36 |  |  |
| Caregivers | | | | | |
|  | Most responsible | 5 | 10 |  |  |
|  | Medium | 39 | 66 | 0.118 (2) | 0.943 |
|  | Least | 24 | 44 |  |  |
|  | Item non-respondents | 10 | 33 |  |  |
| GPs | | | | | |
|  | Most responsible | 50 | 89 |  |  |
|  | Medium | 15 | 32 | 1.836 (2) | 0.399 |
|  | Least | 5 | 4 |  |  |
|  | Item non-respondents | 8 | 28 |  |  |
| HCS | | | | | |
|  | Most responsible | 1 | 8 |  |  |
|  | Medium | 53 | 73 | 5.742 (2) | 0.057 |
|  | Least | 13 | 34 |  |  |
|  | Item non-respondents | 11 | 38 |  |  |
| Pharmacies | | | | | |
|  | Most responsible | 4 | 6 |  |  |
|  | Medium | 54 | 90 | 0.025 (2) | 0.987 |
|  | Least | 12 | 20 |  |  |
|  | Item non-respondents | 8 | 37 |  |  |
| DS retailers | | | | | |
|  | Most responsible | 17 | 16 |  |  |
|  | Medium | 23 | 54 | 4.955 (2) | 0.084 |
|  | Least | 29 | 42 |  |  |
|  | Item non-respondents | 9 | 41 |  |  |

DS, dietary supplement. GP, general practitioner. HCS, home care service. The nurse category may include social educators and other health-related education at bachelor’s level. Nurse assistants include auxiliary nurses, other individuals with health-related education (three years of upper secondary school), and employees without formal education. DS retailers could be health food store staff, internet retailers, complementary and alternative medicine therapists, or others. Differences between subgroups were tested with Chi-square test. Bonferroni adjusted α was 0.05/6 resulting in α=0.008. There were no statistically significant differences between subgroups.

## Suppl. Table 4b. The respondents' ranking of responsibility for the safety of clients with dementia who use DSs. Years of work experience.

|  |  | 0-5 years’ experience | 6-15 years’ experience | <15 years’ experience | *P* |
| --- | --- | --- | --- | --- | --- |
| Persons with dementia themselves | | | | | |
|  | Most responsible | 6 | 1 | 2 |  |
|  | Medium | 16 | 18 | 17 | 0.669 |
|  | Least | 53 | 39 | 33 |  |
|  | Item non-responders | 16 | 16 | 13 |  |
| Caregivers | | | | | |
|  | Most responsible | 5 | 7 | 3 |  |
|  | Medium | 39 | 33 | 33 | 0.800 |
|  | Least | 30 | 21 | 16 |  |
|  | Item non-respondents | 17 | 13 | 13 |  |
| GPs | | | | | |
|  | Most responsible | 44 | 46 | 48 |  |
|  | Medium | 24 | 13 | 10 | 0.081 |
|  | Least | 3 | 4 | 2 |  |
|  | Item non-respondents | 20 | 11 | 5 |  |
| HCS | | | | | |
|  | Most responsible | 3 | 4 | 2 |  |
|  | Medium | 52 | 35 | 38 | 0.899 |
|  | Least | 16 | 18 | 13 |  |
|  | Item non-respondents | 20 | 17 | 12 |  |
| Pharmacies | | | | | |
|  | Most responsible | 5 | 5 | 0 |  |
|  | Medium | 54 | 45 | 44 | 0.595 |
|  | Least | 13 | 9 | 10 |  |
|  | Item non-respondents | 19 | 15 | 11 |  |
| DS retailers | | | | | |
|  | Most responsible | 17 | 10 | 6 |  |
|  | Medium | 28 | 26 | 22 | 0.838 |
|  | Least | 28 | 21 | 22 |  |
|  | Item non-respondents | 18 | 17 | 15 |  |

DS, dietary supplement. GP, general practitioner. HCS, home care service. Years of work experience were given in the ranges: 1-5 years’ experience, 6-15 years’ experience and >15 years’ experience. DS retailers could be health food store staff, internet retailers, complementary and alternative medicine therapists, or others. Differences between subgroups were tested with Fischer’s exact test. Bonferroni adjusted α was 0.05/6 resulting in α=0.008. There were no statistically significant differences between subgroups.

## Suppl. Table 5. Professional practice experience related to DS use by clients with dementia

| **How often do you, as an employee in home care service,** | **Several times a week** | **Weekly-monthly** | **Monthly-**  **bi-annually** | **Bi-annually-**  **annually** | **Annually or**  **less often** | **Never** | **Respondents with experience** | | **Difference in work experience** |
| --- | --- | --- | --- | --- | --- | --- | --- | --- | --- |
|  | **n** | **n** | **n** | **n** | **n** | **n** | **n** | **(%)** | ***P*** |
| Fear that clients might suffer harm due to their DS use  n=213 | 3 | 4 | 17 | 17 | 66 | 106 | 107 | (50) | 0.416 |
| Experience that caregivers raise concern about clients’ DS use  n=222 | 0 | 0 | 1 | 5 | 30 | 186 | 36 | (16) | 0.387 |
| Consult caregivers concerning the safety of clients because of their DS use n=225 | 0 | 0 | 2 | 9 | 29 | 185 | 40 | (18) | 0.242 |
| Experience that clients consult you regarding their DS use  n=227 | 0 | 0 | 3 | 17 | 51 | 156 | 71 | (31) | 0.055 |
| Observe DSs in the homes of clients  n=226 | 6 | 28 | 20 | 36 | 80 | 56 | 170 | (75) | 0.201 |
| Intervene with clients’ DS use to avoid harm to their health n=224 | 0 | 0 | 1 | 9 | 45 | 169 | 55 | (25) | 0.367 |

DS, Dietary supplement. Years of work experience were given in the ranges: 1-5 years’ experience, 6-15 years’ experience and >15 years’ experience. Differences between subgroups were tested with Fisher's exact test. Bonferroni adjusted α was 0.05/6 resulting in α=0.008. There were no statistically significant differences between subgroups.

## Suppl. Table 6. Interventions to increase the safety of clients with dementia who used DS. Years of work experience.

| **Interventions to increase safety** | **Respondents** | **Years of work experience** | | | **Differences** |
| --- | --- | --- | --- | --- | --- |
|  | **n** | **0-5** | **6-15** | **>15** | ***p*** |
|  |  | **n** | **n** | **n** |  |
| Consulted GP  n=71 | 32 | 9 | 9 | 14 | 0.261 |
| Consulted pharmacy  n=70 | 6 | 3 | 1 | 23 | 0.134† |
| Consulted caregiver  n=71 | 19 | 6 | 5 | 8 | 0.667 |
| Asked caregiver to remove DSs  n=70 | 12 | 4 | 4 | 4 | 1.000† |
| Took action to include DSs in automated drug-dispensing system  n=70 | 29 | 10 | 11 | 8 | 0.426 |
| Discussed the problem with colleagues  n=70 | 35 | 14 | 7 | 14 | 0.186 |

GP, general practitioner; DS, dietary supplement. Differences between subgroups were tested with Chi square test or Fischer’s exact test †. Bonferroni adjusted α was 0.05/6 resulting in α=0.008. There were no statistically significant differences between subgroups.
